# Sensory and motor cortical excitability changes induced by rTMS and sensory stimulation in stroke: A randomized clinical trial

**DOI:** 10.3389/fnins.2022.985754

**Published:** 2023-01-25

**Authors:** Aristela de Freitas Zanona, Andressa Claudia Romeiro da Silva, Adriana Baltar do Rego Maciel, Livia Shirahige Gomes do Nascimento, Amanda Bezerra da Silva, Daniele Piscitelli, Katia Monte-Silva

**Affiliations:** ^1^Applied Neuroscience Laboratory, Department of Physical Therapy, Universidade Federal de Pernambuco, Recife, Pernambuco, Brazil; ^2^Occupational Therapy Department and Post-Graduate Program in Applied Health Sciences, Universidade Federal de Sergipe, São Cristóvão, Brazil; ^3^School of Medicine and Surgery, University of Milano-Bicocca, Milan, Italy; ^4^Department of Kinesiology, University of Connecticut, Storrs, CT, United States

**Keywords:** transcranial magnetic stimulation, stroke, somatosensory cortex, occupational therapy, neurorehabilitation, motor evoked potential, somatosensory evoked potential, upper limb

## Abstract

**Background:**

The ability to produce coordinated movement is dependent on dynamic interactions through transcallosal fibers between the two cerebral hemispheres of the brain. Although typically unilateral, stroke induces changes in functional and effective connectivity across hemispheres, which are related to sensorimotor impairment and stroke recovery. Previous studies have focused almost exclusively on interhemispheric interactions in the primary motor cortex (M1).

**Objective:**

To identify the presence of interhemispheric asymmetry (ASY) of somatosensory cortex (S1) excitability and to investigate whether S1 repetitive transcranial magnetic stimulation (rTMS) combined with sensory stimulation (SS) changes excitability in S1 and M1, as well as S1 ASY, in individuals with subacute stroke.

**Methods:**

A randomized clinical trial. Participants with a single episode of stroke, in the subacute phase, between 35 and 75 years old, were allocated, randomly and equally balanced, to four groups: rTMS/sham SS, sham rTMS/SS, rTMS/SS, and sham rTMS/Sham SS. Participants underwent 10 sessions of S1 rTMS of the lesioned hemisphere (10 Hz, 1,500 pulses) followed by SS. SS was applied to the paretic upper limb (UL) (active SS) or non-paretic UL (sham SS). TMS-induced motor evoked potentials (MEPs) of the paretic UL and somatosensory evoked potential (SSEP) of both ULs assessed M1 and S1 cortical excitability, respectively. The S1 ASY index was measured before and after intervention. Evaluator, participants and the statistician were blinded.

**Results:**

Thirty-six participants divided equally into groups (nine participants per group). Seven patients were excluded from MEP analysis because of failure to produce consistent MEP. One participant was excluded in the SSEP analysis because no SSEP was detected. All somatosensory stimulation groups had decreased S1 ASY except for the sham rTMS/Sham SS group. When compared with baseline, M1 excitability increased only in the rTMS/SS group.

**Conclusion:**

S1 rTMS and SS alone or in combination changed S1 excitability and decreased ASY, but it was only their combination that increased M1 excitability.

**Clinical trial registration:**

clinicaltrials.gov, identifier (NCT03329807).

## Background

The ability to produce coordinated movement is dependent on dynamic interactions through transcallosal fibers between the two cerebral hemispheres of the brain (Gerloff and Andres, 2002). Although typically unilateral, stroke induces changes in functional and effective connectivity across hemispheres, which are related to sensorimotor impairment and stroke recovery (Rehme and Grefkes, 2013; Brito et al., 2021).

Interhemispheric interaction in the primary motor cortex (M1) has been widely studied (Jang, 2010; Lindenberg et al., 2012). Following a stroke, an imbalance of interhemispheric interaction between motor areas has been observed because of decreased excitability in M1 of the lesioned hemisphere and increased excitability in the non-lesioned hemisphere (Nowak et al., 2009) [reviewed in Rossini et al. (2003)]. This functional organization is the underlying hypothetical model that supports the use of non-invasive brain stimulation therapies for increasing M1 excitability of the lesioned hemisphere and decreasing M1 excitability of the non-lesioned hemisphere (Du et al., 2019). Within this model, the rebalance of interhemispheric interactions may enhance motor function recovery in post-stroke individuals (Cramer and Crafton, 2006; Rossini et al., 2007). Previous studies have focused almost exclusively on interhemispheric interactions in M1 (Murase et al., 2004), while the imbalance of interaction between the sensory areas remains unknown (Calautti et al., 2007).

The primary somatosensory cortex (S1) is involved in the integration of multimodal information through connections with M1. Changes in S1 activity and sensory networks are known to be involved in motor learning; therefore, the integrity of sensory cortex connectivity may be an essential marker of post-stroke motor function (Frias et al., 2018).

Several clinical trials have suggested that sensory stimulation (SS) facilitates functional reorganization of M1 (Hamdy et al., 1998; Garry et al., 2005; Ridding and Ziemann, 2010) and promotes motor recovery (Brodie et al., 2014a). Similarly, the application of repetitive transcranial magnetic stimulation (rTMS) over S1 enhances motor learning in patients with chronic stroke individuals (Brodie et al., 2014a). Previous studies using high-frequency rTMS over S1 demonstrated increased M1 excitability in healthy individuals (Rizzo et al., 2004; Boros et al., 2008), suggesting that S1 stimuli may change M1 excitability. However, the neurophysiological mechanisms subjacent the effects of somatosensory stimulation are still not fully understood. Insights into how rTMS (i.e., central somatosensory stimulation) and SS (i.e., peripheral stimulation), when applied alone or in combination, act on cortical excitability of M1 and S1 might help in the development of more effective and efficient therapies.

Thus, this study aimed to investigate the presence of interhemispheric asymmetry (ASY) in S1 and to observe whether the application of rTMS over S1 alone or in combination with SS, modulates M1 and S1 excitability and S1 ASY in subacute post-stroke individuals. While the majority of previous studies were conducted in acute and chronic stroke, the present study addresses the subacute stroke phase. Such a time window represents an appropriate time for rehabilitation to enhance and guide optimal spontaneous reorganization of sensorimotor networks, thus facilitating the functional recovery process.

Similar to M1, we hypothesized that interhemispheric asymmetry could also occur in S1 and that the combination of central and peripheral stimulation is superior to monotherapy. We expected that combining rTMS with other therapies, such as SS, could optimize the plastic effects induced by multisensory stimulation and lead to more significant changes in sensory and motor cortical excitability.

## Materials and methods

### Study design

This research is part of a randomized and triple-blind clinical trial duly registered in clinicaltrials.gov (NCT03329807) and approved by the local research ethics committee (69908217.7.0000.5208). The research was conducted at the Laboratory of Applied Neuroscience of the Federal University of Pernambuco, Recife, Brazil.

### Participants

The inclusion criteria were as follows: participants aged between 30 and 75 years with a diagnosis of ischemic or hemorrhagic stroke in the subacute phase [3–24 weeks, see Bernhardt et al. (2017)] and an upper limb Fugl-Meyer Assessment (FMA) motor score (0–66 points) between 10 and 62 and sensory score (0–12 points) between 2 and 10. Participants were excluded if they had cognitive deficits [Mini Mental State Examination–MMSE score<18–(Folstein et al., 1975)] or a history of multiple brain lesions, other associated neurological diseases, peripheral sensory disorders, or a history of psychiatric disorders, including drug and alcohol abuse. Participants who were unable to perceive transcutaneous electrical neurostimulation (TENS) at the hand and forearm, were undergoing concurrent treatment for the upper limb, had rTMS contraindications, and were using medication likely to influence cortical excitability were also excluded (Rossi et al., 2009). Participants with complete anesthesia were also excluded. The sample was selected for convenience.

### Randomization and blinding

The participants were randomized and allocated to four groups of equal size: (i) active rTMS and sham SS (rTMS/sham SS), (ii) sham rTMS and active SS (sham rTMS/SS), (iii) active rTMS and active SS (rTMS/SS), and (iv) sham rTMS and sham SS. A stratified block allocation based on stroke onset and age was generated at by an independent researcher and packed into sequentially numbered, opaque sealed envelopes. A researcher who did not participate in the evaluations or interventions generated the random allocation sequence, enrolled participants, and assigned participants to the interventions. Those evaluating and analyzing the outcomes and participants were blinded to the treatment arm. We stratified the sample by age and stroke time because they could interfere with the interpretation of the effects of treatment outcomes.

### Therapeutic interventions

**FIGURE 1:**
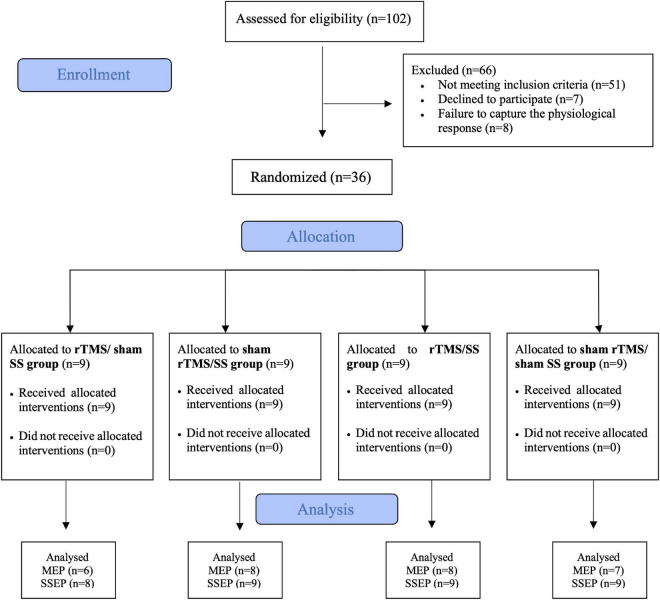
CONSORT flowchart of the study. MEP, motor evoked potential; SSEP, somatosensory evoked potential; SS, sensory stimulation; rTMS, repetitive transcranial magnetic stimulation.

Each therapeutic session lasted 80 min. During the first 20 min, participants were subjected to rTMS (active or sham) (Fregni et al., 2006; Cho et al., 2017) followed by 60 min SS (active or sham). Daily sessions were held over 2 weeks (10 sessions). The intervention was conducted by a researcher not involved in any of the evaluation or randomization procedures, thus ensuring the blinding of study allocation (see Figure 1–Flowchart of the trial design).

#### Repetitive transcranial magnetic stimulation (rTMS)

The rTMS pulses were delivered through a 70 mm 8-shaped coil connected to a Magstim super rapid stimulator (Magstim Co., United Kingdom) over S1. The somatosensory cortex (S1) is 3 cm posterior to C3/C4. Thus, C3/C4 are the 10–10 EEG system referring to M1. The hotspot was defined as the location where the largest and most consistent visual responses were elicited by single pulse TMS for the FDI muscle. Note that, if the FDI hotspot on the lesioned hemisphere was not found, S1 was delimited at a point 3 cm posterior to primary motor cortex located by C3/C4 of 10–20 EEG system. Previous studies had shown changes in sensory function when non-invasive brain stimulation was applied 3 cm posteriorly from the hand area of the primary motor cortex (Fiorio and Haggard, 2005; Koch et al., 2006).

Repetitive TMS [10 Hz, 1,500 pulses; 120% of the resting motor threshold (RMT) for FDI of the non-lesioned hemisphere] was applied over the S1 lesioned hemisphere. The stimulation protocol was defined established on the expected cortical facilitatory effects of high frequency rTMS (Lefaucheur et al., 2020). As previously done by other authors (Wupuer et al., 2013; Milot et al., 2019), the non-lesioned hemisphere RMT was used to determine rTMS intensity. A substantial portion of M1 or corticospinal tract is usually damaged after a stroke and make RMT of lesioned hemisphere increases substantially, being probable that the stimulator output would not have reached 120% RMT for all subjects.

For sham-rTMS, two coils were used. One coil disconnected from the stimulator was placed over S1. The TMS stimulator was discharged through another coil connected to the stimulator and positioned behind the participant’s head (out of his/her view). Thus, while no magnetic pulses were delivered to the participant, they were exposed to acoustic stimulation of the active protocol (Dos Santos et al., 2019; Mendonca et al., 2021).

#### Sensory stimulation

Participants in the active SS group underwent a sensory therapy protocol that consisted of 15 min of active sensory training and 45 min of mirror therapy concomitant with peripheral nerve sensory stimulation (TENS).

The active sensory training protocol was adapted from Carey et al. (2011) and divided into (i) texture discrimination task, employed graded stimuli with various sensory features (texture, shape, size, weight, and hardness), (ii) graphesthesia, (iii) limb position sense task, and (iv) tactile object recognition.

Four different textures and types of objects were used for active sensory training (i). Initially (first stage), the participants were asked, with eyes open and using the non-paretic hand, to identify four characteristics (palpable) of each object (e.g., size, shape, temperature, details, and length of each object). Then, in the second stage, they were encouraged to notice the same four characteristics using the paretic hand. In the third and fourth stages, the tasks of the first and second stages were repeated with the eyes closed. In addition, the participants discriminated textures through a tactile memory game. For graphesthesia exercises (ii), the occupational therapist (OT) asked patients to identify a series of numbers, letters, and geometric shapes that were drawn on the palmar and dorsal surfaces of the hand using a pencil. For the limb position sense task (iii), the paretic upper limb was moved to a position in the flexion-extension, abduction-adduction, and pronation-supination ranges, and the participants indicated the perceived limb position. For tactile object recognition (iv), participants wearing a blindfold had to recognize objects chosen by the OT from a basket placed in front of them. Active sensory training was performed in the non-paretic hand for the sham SS groups.

For the mirror therapy, the paretic upper limb was hidden behind a mirror (50 × 50 cm), and the non-paretic upper limb was placed in front of the mirror. Participants were asked to look at the non-paretic upper limb reflected in the mirror and observe its movements (flexion-extension of the wrist, elbow, and fingers; and pronation-supination of the forearm) (Cho and Cha, 2015). In the sham SS group, non-paretic upper limb movements were performed without a mirror.

Transcutaneous electrical neurostimulation was applied to the median nerve at the wrist of the paretic hand. The cathode was placed 20 mm proximal to the anode. Five electrical pulses (1 ms duration for each) at 10 Hz were delivered every second over 45 min (Conforto et al., 2010) by an electrical stimulator (Model Quark–Dualpex 961). Stimulus intensity was set at the level at which individuals reported mild paresthesia in the nerve territories without pain or visible muscle contraction. The participants were instructed not to perform active muscle contractions during the intervention. For the sham stimulation, the device was turned off 60 s after stimulation onset. TENS was administered concurrently with mirror therapy.

### Outcome measures

#### Motor evoked potential–MEP

The single-pulse TMS-induced MEP of the paretic upper limb was used to assess cortical excitability of the lesioned M1. First, an “8” shaped coil connected to a single-pulse magnetic stimulator (NeuroMS, Neurosoft, Russia) was positioned over the non-lesioned M1 to determine the FDI hotspot of the non-paretic hand. The RMT of the non-lesioned M1 was measured using Motor Threshold Assessment Tool, version 2.0 (Awiszus and Borckardt, 2011). Different from the RMT measurement for rTMS, since the single-pulse magnetic stimulator was connected to a two-channel digital electromyography (EMG; NeuroMep Micro, Neurosoft, Russia), the RMT for the MEP was defined as the minimum stimulus intensity that produced a peak-to-peak amplitude of 50 μV in the FDI muscle during rest, as observed by EMG recordings.

In the present work, the hotspot of the affected hemisphere was found to identify the best place for rTMS therapy. For RMT during single-pulse TMS assessment, the hotspot on the unaffected side was determined because a substantial portion of M1 or corticospinal tract is usually damaged after a stroke making RMT of the injured hemisphere substantially increased.

For MEP of the lesioned hemisphere, the coil was moved and positioned over lesioned M1 hand representation at the FDI hotspot or at C3/C4 position when the FDI hotspot was not found.

The coil positions were marked over the patient’s scalp with washable non-toxic pencils to guarantee identical positions throughout the study. For the analysis, the mean peak-to-peak MEP amplitude of 20 consecutive stimuli at 120% of RMT was used as the motor cortical excitability. Motor cortex excitability was recorded at baseline and after treatment sessions.

#### Somatosensory evoked potential–SSEP

Through the electrical stimulator (Neuro-MEP, Neurosoft), percutaneous stimuli (square pulse, 0.2 ms, 2 mA, 1,000 pulses in total, 3 Hz, distal cathode, and proximal anode) were applied to the median nerve bilaterally with the patient lying down. The somatosensory evoked potential (SSEP) was recorded by EEG surface electrodes positioned at CP3, and CP4 (electroencephalogram 10–20 marking system). Reference electrode was placed in the FPz, and grounding electrodes were placed in anterior neck, below the prominence of the hyoid bone (PA), and in the posterior neck, as well as above the C7 vertebra (PP) (Nuwer et al., 1994). Only the amplitudes of N20 and P23 within 50 ms window were analyzed to investigate the primary somatosensory thalamocortical excitability. The analysis was performed only on pre- and post-intervention amplitudes. The electrode impedance was maintained below 7 kilohms (Kω). A 25–3,000 Hz bandpass filter was applied. NEUROMEP software was used to test for short-latency somatosensory evoked potentials (version 3.7.3.8) to capture and analyze SSEP waves (Cruccu et al., 2008). For the analysis, 1,000 responses were averaged.

The components examined were the SSEP peak-to-peak amplitudes (μV) of components N20 and P23. N20 originated from the somatosensory thalamus-cortical radiation, and P23 originated from potential postsynaptic graduates, both generated within the primary somatosensory cortex. For brain injuries, a decrease in the N20/P23 amplitude is expected (Luccas et al., 1990). The results of the amplitudes of the patients who did not manifest responses capable of being captured by the software algorithm were not subjected to SSEP analysis. The recordings were made only before the beginning of the interventions and immediately after the end of the 10 sessions.

### Data processing and analysis

Statistical analysis was performed using non-parametric tests since the variables were non-normally distributed (Shapiro–Wilk test, *p* > 0.05). A Kruskal–Wallis test for continuous variables and a Fisher’s exact test for categorical variables were used to analyze differences in baseline characteristics among the groups. The data are presented as median [interquartile range, IQR] unless otherwise specified.

For SSEP, an index of asymmetry between the non-lesioned and lesioned hemispheres was calculated for each group. Interhemispheric asymmetry (ASY) was calculated using the ratio between the non-lesioned/lesioned hemispheres. Thus, an ASY greater than 1 indicates increased non-lesioned sensory cortical excitability relative to lesioned excitability.

For MEP, the baseline and post-treatment MEPs were normalized intra-individually and were given as baseline ratios. Thus, a ratio greater than 1 indicates increased cortical excitability, whereas a ratio less than 1 indicates decreased excitability.

For all measurements, the Kruskal–Wallis test was used to assess differences among groups. Mann–Whitney and Wilcoxon tests were used for between-group and within-group comparisons (not adjusted for multiple comparisons).

For MEPs, the sample size was calculated based on findings of a pilot study on MEPs amplitude (effect size = 1.7) with an α = 0.05 and power (β) of 0.85. Therefore, an estimated total sample size of 32 subjects, with a minimum of 8 subjects for each group, was considered sufficient. For SSPE, based on a β = 0.85, with α = 0.05 and an effect size = 1.9, a total sample size of 28 subjects, with a minimum of 7 subjects per group, was required. Sample size was computed in G*Power software (Faul et al., 2007).

To investigate the robustness of overall finding, the effect size (d) using Cohen’s d was calculated. According to Cohen (1992) *d* = 0.2 is a small treatment effect, *d* = 0.5 represents a moderate effect, and *d* = 0.8 is a large effect. All analyses were performed using Statistical Package for Social Sciences (SPSS) version 18 software. A significance level of *p* ≤ 0.05 was adopted.

## Results

The descriptive demographic and clinical data of participants are presented in Table 1. No significant differences among the groups were found at baseline.

**TABLE 1:** Demographic and clinical characteristics for each group at baseline.

	rTMS/sham SS (*n* = 9)	sham rTMS/SS (*n* = 9)	rTMS/SS (*n* = 9)	sham rTMS/sham SS (*n* = 9)	*P*-value
Age median (IQR)	63 (59.7 to 75)	66.5 (57.7 to 72)	63.6 (60 to 72)	63 (59 to 75)	0.998^2^
Gender, male *n* (%)	4 (44.4)	5 (55.5)	4 (44.4)	3 (33.3)	0.708^1^
Type stroke, ischemic *n* (%)	9 (100)	9 (100)	9 (100)	9 (100)	0.526^1^
Stroke time (weeks) median (IQR)	8.5 (5 to 22)	8 (4.7 to 20)	11 (8 to 22)	11.5 (6.2 to 22)	0.601^2^
Hemiparesis, right n (%)	4 (44.4)	3 (33.3)	5 (55.5)	6 (66.6)	0.424^1^
Dominance, right n (%)	9 (100)	8 (88.8)	9 (100)	9 (100)	0.428^1^
MMSE median (IQR)	25.5 (24 to 30)	25.5 (24 to 30)	24 (21.2 to 29)	19.5 (18 to 30)	0.109^2^
FM-Motor median (IQR)	53.5 (40.2 to 62)	37.5 (19.5 to 60)	48.5 (39.2 to 60)	36.5 (29.7 to 61)	0.422^2^
FM-Sensory median (IQR)	9.5 (7.5 to 10)	7 (5.2 to 10)	8 (7 to 10)	8 (6 to 8.7)	0.514^2^

FM-M, Fugl-Meyer; MMSE, Mini Mental State examination score; SS, peripheral somatosensory stimulation; rTMS, repetitive transcranial magnetic stimulation. Data are presented as median, interquartile range, absolute frequency and relative frequency.

^1^Chi-square test and ^2^ Kruskal-Wallis.

Seven patients (three from rTMS/sham SS, one from sham rTMS/SS, one from rTMS/SS, and two from sham rTMS/sham SS group) were excluded from MEP analysis because of failure to produce consistent MEP at 120% of RMT. One participant was excluded from the rTMS/sham SS group in the SSEP analysis because no SSEP was detected. All participants completed 10 sessions (Figure 1).

As detailed in Table 2, at baseline, an ASY in S1 (SSEP of non-paretic UL vs. paretic UL) was observed for all groups (Wilcoxon test, *p* < 0.05). Baseline SSEP amplitudes did not differ among the groups (Kruskal–Wallis test, *x*^2^ = 2.56, *p* = 0.464). After treatment, an ASY was observed only in the sham rTMS/Sham SS (Table 2).

**TABLE 2:** Somatosensory evoked potential of both upper limbs (non-paretic and paretic) before (baseline) and after treatment (post-treatment) for each group.

Groups	Baseline			Post-treatment		
	Non-paretic UL (μ V)	Paretic UL (μ V)	ASY	Non-paretic UL (μ V)	Paretic UL (μ V)	ASY
rTMS/sham SS	3.84 (1.1 to 7.9)	0.89 (0.7 to 5.5)	3.04*	2.46 (0.9 to 7.9)	1.79 (0.7 to 6.1)	0.76
sham rTMS/SS	4.36 (1.8 to 8.4)	0.89 (0.3 to 3.5)	3.56*	2.88 (1.7 to 8.4)	1.21 (0.6 to 6.7)	1.67
rTMS/SS	2.88 (1.5 to 7.8)	0.39 (0.1 to 4.9)	2.58*	2.16 (0.8 to 6.1)	1.12 (0.2 to 6.8)	1.04
sham rTMS/Sham SS	5.53 (4 to 7.6)	3.03 (0.5 to 4.4)	2.53*	4.53 (3.5 to 7.2)	2.40 (0.1 to 3.4)	2.13*

ASY, interhemispheric asymmetry; SS, peripheral somatosensory stimulation; rTMS, repetitive transcranial magnetic stimulation; UL, upper limb. Data are presented median and interquartile range.

*p<0.05, Wilcoxon tests.

**FIGURE 2:**
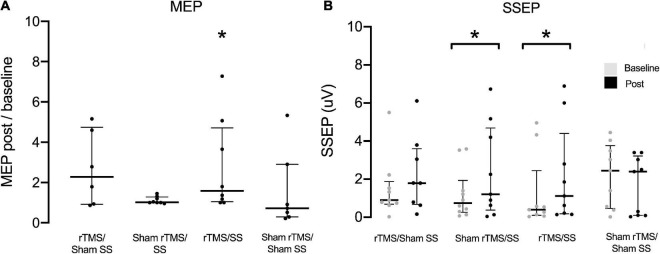
Motor evoked potential (MEP) of lesioned hemisphere before (baseline) and after treatment for each group **(A)** and Somatosensory evoked potential (SSEP) of paretic upper limbs **(B)**. In **(A)**, MEP data were normalized intraindividually and shown as baseline ratios. μV, Microvolts; SS, peripheral somatosensory stimulation; rTMS, repetitive transcranial magnetic stimulation. Data are presented as median and interquartile range *p < 0.05. Wilcoxon test (baseline Vs. post-treatment).

Figure 2 shows the SSEP and MEP for the paretic UL at baseline and post-intervention. Compared with baseline, the paretic UL SSEP amplitudes increased in Sham rTMS/SS [0.74 (0.26 to 1.9) Vs. 1.2 (0.38 to 4.7), Wilcoxon test *p* = 0.046, *d* = 0.6] and rTMS/SS [0.39 (0.1 to 2.5) Vs. 1.1 (0.18 to 4.4), Wilcoxon test *p* = 0.025, *d* = 0.4], but not for the rTMS/sham SS group [0.9 (0.69 to 1.9) Vs. 1.8 (0.67 to 3.6) Wilcoxon test *p* = 0.075,*d* = 0.3] and the sham rTMS/sham SS group [2.4 (0.47 to 3.8) Vs. 2.4 (0.09 to 3.2) Wilcoxon test *p* = 0.107, *d* = 0.3] (Figure 2). No differences were found between the groups (Kruskal–Wallis test, *p* > 0.05). No significant changes were observed in the SSEP of non-paretic UL post-treatment when compared to baseline (Wilcoxon test, *p* > 0.05).

A significant increase in MEP amplitudes in M1 was found after interventions only for the rTMS/SS group: 1 Vs. 1.59 [1.05 to 4.72], Wilcoxon test *p* = 0.028. No differences were found in the rTMS/sham SS: 1 Vs. 2.29 [0.9 to 4.74], Wilcoxon test *p* = 0.116; sham rTMS/SS: 1 Vs. 1.02 [1.0 to 1.28], Wilcoxon test *p* = 0.138, and sham rTMS/Sham SS groups: 1 Vs. 0.72 [0.29 to 2.9], Wilcoxon test *p* = 0.866. No differences in the between-groups analysis were observed at baseline and after the intervention (Kruskal–Wallis test, *p* > 0.05) (Figure 2). The RMT values of non-lesioned hemisphere remained stable before and after the intervention (see Supplementary Figure 1, Bland-Altman plots).

## Discussion

In summary, our findings indicated interhemispheric asymmetry of the primary somatosensory cortex after stroke at baseline, which was normalized after somatosensory stimulation. All somatosensory stimulation modalities increased S1 excitability, but only combined therapy (rTMS/SS) modulated M1 excitability.

### S1 interhemispheric asymmetry

Similar to the motor system, previous studies have pointed out that activation of S1 in one hemisphere increased inhibition from activated sensory areas toward homologous areas of the contralateral hemisphere, suggesting the existence of interhemispheric inhibitory interactions between S1 in human participants (Hlushchuk and Hari, 2006; Blankenburg et al., 2008; Eickhoff et al., 2008; Kastrup et al., 2008; Klingner et al., 2011). Maladaptive functioning of interhemispheric inhibition in M1 has been described in patients with stroke and probably influences functional recovery in these patients (Murase et al., 2004). Assuming that interhemispheric inhibition also occurs between S1 (Brodie et al., 2014b), we expected the existence of an S1 interhemispheric asymmetry after stroke. Indeed, our findings demonstrated S1-S1 asymmetry in all groups before the intervention. The transcallosal disinhibition hypothesis described in M1 (Rossini et al., 2003; Murase et al., 2004; Nowak et al., 2009) could also explain the S1 interhemispheric asymmetry. Following a stroke in the primary somatosensory cortex, S1 cortical excitability of the lesioned hemisphere would be decreased due to the infarct and S1 of the contralesional hemisphere is disinhibited, thus leading to enhanced inhibition toward S1 of the lesioned hemisphere (Frias et al., 2018).

Future studies may verify the relationship between S1 interhemispheric asymmetry and motor and sensory impairments in this population.

### S1 and M1 cortical excitability changes after central and peripheral somatosensory stimulation

Normalization of interhemispheric asymmetry after stroke has been associated with functional sensorimotor recovery (Cramer and Crafton, 2006; Rossini et al., 2007). However, faced with the opposite theories of central nervous system reorganization after stroke (Di Pino et al., 2014), the normalization of the interhemispheric imbalance may be a too simplified approach to fit for all stroke patients with different levels of sensorimotor severity.

The increased excitability in S1 of the lesioned hemisphere after somatosensory stimulation may have contributed to improvement in the symmetry between excitability and functioning of both cortices. SS activates afferent tracts terminating in the contralateral thalamus, which in turn mainly forward the SI on the postcentral gyrus of the contralateral hemisphere, thus resulting in increased S1 excitability (Kaas, 2004). Indeed, as revealed by functional magnetic resonance imaging (fMRI) and electroencephalography (EEG) studies, peripheral stimulation in one hand is associated with enhanced neural activity in predominantly contralateral somatosensory areas in healthy individuals (Nihashi et al., 2005; Sutherland and Tang, 2006).

Also, by central somatosensory stimulation (5 Hz rTMS applied over the S1) Ragert et al. (2004) R1.24 induced sustained increases in S1 cortical excitability, analyzing it by a paired-pulse protocol consisting of paired electrical stimulation of the median nerve in combination with recordings of SEEPs, in healthy individuals.

A similar effect was also observed when intermittent theta burst stimulation (iTBS), an excitatory form of patterned rTMS, was applied over S1 (Katayama and Rothwell, 2007; Premji et al., 2010). Although, the precise mechanisms mediating the effects of rTMS on cortical excitability, either on M1 or S1, are still unclear, it has been proposed that rTMS influences Na + and Ca + + channels and NMDA-receptor activity (Valero-Cabre et al., 2017).

Given the somatosensory system has strong structural and functional connections with the motor system (Petreanu et al., 2009; Xu et al., 2012; Borich et al., 2015) as well as the current study’s findings of increased S1 excitability of the lesioned hemisphere after the interventions, we expected that rTMS and SS, either alone or combined, would also increase M1 excitability. In contrast to previous studies using a 2-h period of somatosensory stimulation (Kaelin-Lang et al., 2002; Ridding and Ziemann, 2010) and using 30 Hz-rTMS (Jacobs et al., 2014), SS and rTMS over S1 alone did not increase MEP amplitudes in our study. The longer duration of peripheral stimulation and higher rTMS frequency in previous studies could explain these distinct neural responses. Indeed, evidence suggests that rTMS can modulate cortical excitability in a frequency-and intensity-dependent manner (Siebner and Rothwell, 2003; Ridding and Ziemann, 2010). Additional experiments are required to gain additional insights into this issue.

Few studies have focused on association of rTMS with peripheral stimulation for modulating S1 excitability. Previous studies focused on combination of rTMS with peripheral stimulation for the modulation of M1 excitability. We hypothesized that, similar to what happens in motor stimulation, the synchronous application of both forms of sensory stimulation (peripheral and central) could potentially enhance the effect of each therapy alone. Previous studies supported the advantages of associating motor therapies with rTMS over M1 (Rose et al., 2014; Hosomi et al., 2016; Tosun et al., 2017; Du et al., 2019). The combination of cortical stimulation over M1 with peripheral sensory stimulation was also found to be a promising strategy in facilitating motor function after stroke better than performing each technique in isolation (Celnik et al., 2009).

The use of different sensory strategies has been encouraged in recent studies to treat sensorimotor deficits. Indeed, multisensory stimulation through exposure to an enriched environment increases brain plasticity and recovery of function after stroke (Bolognini et al., 2015; Tinga et al., 2016; Hakon et al., 2018; Sathian and Ramachandran, 2020).

While it is possible to expect that the active sensory training with the non-paretic upper limb could modulate contralesional S1 excitability and may confound the interpretation of our results, no significant changes were found in the sham rTMS/sham SS group. Notably the SSEP tended to increase in the non-lesioned hemisphere, however these changes were not statistically significant, indicating that the active sensory training performed in the non-paretic hand of our protocol was ineffective to interfere with sensory brain activity.

### Implications for rehabilitation

Stroke might significantly modify the interhemispheric symmetry of the sensorimotor cortex (Nowak et al., 2009). Studies suggest that an interhemispheric imbalance of motor cortices post-stroke is positively associated with the severity of paretic hand impairment (Murase et al., 2004) and likely interferes with recovery (Calautti et al., 2007; Tang et al., 2015). Considering that impairment in somatosensory structures and function may also contribute to motor disability (Borich et al., 2015; Piscitelli et al., 2020), we expected S1 excitability asymmetry to be associated with motor impairment. Indeed, previous studies have demonstrated that abnormal interhemispheric connectivity between the primary sensory cortices is associated with motor impairment after stroke (Frias et al., 2018). This leads to the hypothesis that “rebalancing” of interhemispheric symmetry of the sensorimotor cortex in patients with stroke by rTMS might promote improvement of upper limb function. Future studies should consider investigating the relationship between impairments in somatosensory areas and functional outcomes.

### Limitations

Due to the small sample size, our study is considered a pilot study. Results, should be treated with cautions as *post-hoc* tests were not corrected for multiple comparisons, thus remain exploratory. Studies with larger sample sizes are needed to replicate our results and validate our conclusions. We also acknowledge that the patients who were excluded from the MEP and SSEP analyses and other control variables that may influence cortical excitability were limitations of this study. Another limitation of the current study is that we did not directly assess M1 cortical excitability in the non-lesioned hemisphere to verify the relationship between M1-M1 and S1-S1 asymmetry. Although implications for clinical practice have been discussed, a limitation of this study was the lack of correlation analysis between cortical excitability changes and functional outcomes [sensory and motor function data were published earlier in De Freitas Zanona et al. (2022)]. Finally, another limitation concerns the group that performed the sham sensory stimulation on the unaffected limb. In fact, active sensory training performed with the non-paretic upper limb could modulate contralesional S1 excitability. However, the amplitude of somatosensory evoked potentials did not change for the sham rTMS-sham SS group which seem point out to the active sensory training performed in the non-paretic hand of our protocol was ineffective to interfere with sensory function. Future studies should consider investigating the relationship between impairments in somatosensory areas and functional outcomes.

## Conclusion

Our results demonstrated that somatosensory stimulation (rTMS and SS alone or in combination) reduces S1 interhemispheric asymmetry in patients with subacute stroke. This reduction in S1-S1 asymmetry is concurrent with enhanced S1 excitability. In addition, based on the findings of cortical M1 excitability, we found that rTMS may enhance the effects of SS. Further research is needed to investigate the effects of combined therapies on stroke rehabilitation.

## Data availability statement

The raw data supporting the conclusions of this article will be made available by the authors, without undue reservation.

## Ethics statement

The studies involving human participants were reviewed and approved by Ethics and Research Universidade Federal de Pernambuco Committee (69908217.7.0000.5208). The patients/participants provided their written informed consent to participate in this study.

## Author contributions

AF and KM-S designed the study and wrote the manuscript. AF, AR, and ABdRM collected data. ABdS and LS analyzed and interpreted the data and performed statistical analysis. KM-S supervised the study. DP helped with data interpretation and manuscript drafting. All authors approved the final version of the manuscript read and approved the final manuscript.

## Supplementary material

Supplementary Figure 1 Bland-Altman plots of resting motor threshold (RMT) values of non-lesioned hemisphere before and after treatment. SS, sensory stimulation; rTMS, repetitive transcranial magnetic stimulation.
